# Persistent HIV-1 transcription during ART: time to reassess its significance?

**DOI:** 10.1097/COH.0000000000000849

**Published:** 2024-03-12

**Authors:** Céline Fombellida-Lopez, Ben Berkhout, Gilles Darcis, Alexander O. Pasternak

**Affiliations:** a Laboratory of Experimental Virology, Department of Medical Microbiology, Amsterdam UMC, University of Amsterdam, Amsterdam, The Netherlands; b Laboratory of Immunology and Infectious Diseases, GIGA-Institute, University of Liège; c Department of General Internal Medicine and Infectious Diseases, University Hospital of Liège, Liège, Belgium

**Keywords:** cell-associated HIV-1 RNA, defective proviruses, HIV-1 cure, HIV-1 reservoir, immune activation, residual HIV-1 pathogenesis

## Abstract

**Purpose of review:**

Despite suppressive antiretroviral therapy (ART), HIV-1 reservoirs persist and reignite viral replication if therapy is interrupted. Persistence of the viral reservoir in people with HIV-1 (PWH) is the main obstacle to an HIV-1 cure. The reservoirs are not transcriptionally silent, and viral transcripts can be detected in most ART-treated individuals. Here, we review the recent progress in the characterization of persistent HIV-1 transcription during ART.

**Recent findings:**

Evidence from several studies indicates that, although cell-associated unspliced (US) HIV-1 RNA is abundantly expressed in ART-treated PWH, intact full-length US transcripts are rare and most US RNA is derived from defective proviruses. The transcription- and translation-competent defective proviruses, previously considered irrelevant, are increasingly being linked to residual HIV-1 pathogenesis under suppressive ART. Recent data suggest a continuous crosstalk between the residual HIV-1 activity under ART and the immune system. Persistent HIV-1 transcription on ART, despite being mostly derived from defective proviruses, predicts viral rebound upon therapy interruption, suggesting its role as an indicator of the strength of the host antiviral immune response that is shaping the viral rebound.

**Summary:**

In light of the recent findings, the significance of persistent HIV-1 transcription during ART for the long-term health of PWH and the cure research should be reassessed.

## INTRODUCTION

In 2022, an estimated 39 million people worldwide were living with the human immunodeficiency virus type 1 (HIV-1). Among them, 29.8 million people (76%) were accessing antiretroviral therapy (ART) (Global HIV & AIDS statistics, Fact sheet, UNAIDS; https://www.unaids.org/en/resources/fact-sheet). By targeting essential stages of HIV-1 replication cycle, ART suppresses viral replication, preserves immune function and prevents the development of acquired immunodeficiency syndrome (AIDS) [1]. People with HIV-1 (PWH) who adhere to ART typically experience improved health and a life expectancy that is only a few years shorter than that of uninfected individuals [2]. Nevertheless, despite the undeniable success of ART, it is not curative and has to be taken lifelong. The primary challenge in achieving a cure for HIV-1 lies in the establishment, in the early course of infection, and lifelong persistence of the viral reservoir, located in resting memory CD4+ T cells and other cell types, primarily in peripheral blood and lymphoid organs [3–5]. The HIV-1 reservoir decays so slowly over time [6,7] that its lifelong persistence is guaranteed in the absolute majority of cases. Such long-term persistence is due to integration of the viral genome into the host chromosome as part of HIV-1 replication cycle. Once integrated, the provirus persists for the lifetime of the cell, and division of an infected cell provides each progeny cell with a copy of the integrated provirus. The reservoir persists primarily through cellular longevity and proliferation [8–12], although replenishment by low-level residual virus replication, for example due to limited penetration of antiretroviral drugs into tissues, has been suggested to contribute as well [13–15].

Several excellent reviews have summarized our current knowledge of HIV-1 reservoirs in PWH on ART [16–19]. Traditionally, the reservoir has been viewed as integrated proviruses that are transcriptionally silent and persist essentially as the viral genetic code but can be reactivated to transcribe viral RNA and produce infectious virus. In the recent years, however, it has become clear that transcriptional latency is not the only, and maybe even not the main, mechanism of HIV-1 persistence, as the reservoir is far from being silent [20–24]. Multiple studies have detected cell-associated (CA) HIV-1 RNA in the majority of peripheral blood and tissue samples from PWH on prolonged ART in the absence of *ex vivo* stimulation [25–29]. Estimates of the relative size of the active reservoir (percentage of persistent proviruses that transcribe HIV-1 RNA) vary between studies [30,31,32^▪▪^,^33^^▪▪^], but it is clear that a substantial part of the HIV-1 reservoir is not transcriptionally silent. Here, we discuss some recent insights into the significance of this persistent HIV-1 transcription during ART and its potential implications for the cure research. 

**Box 1:**
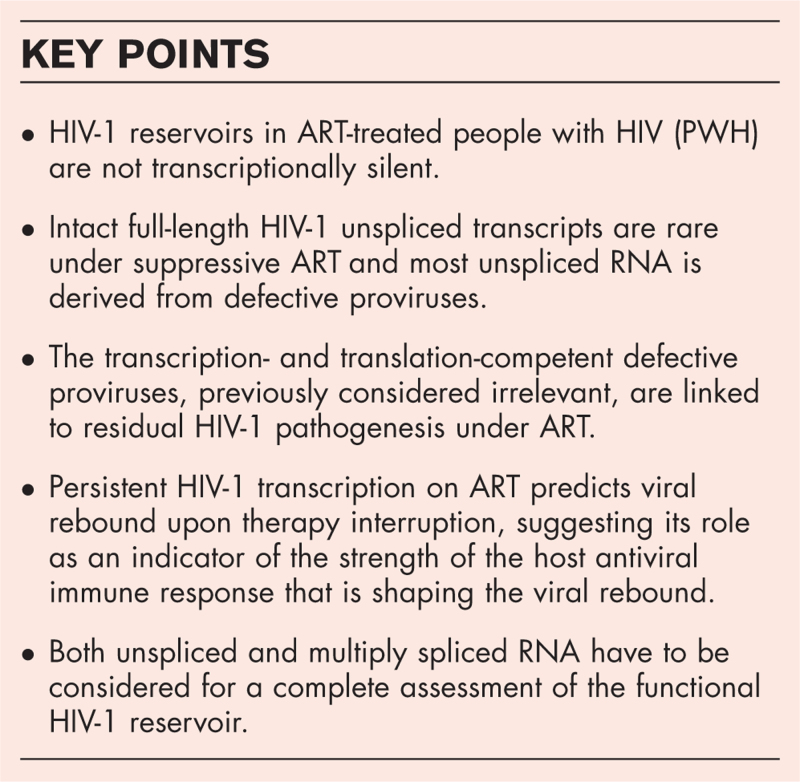
no caption available

## HIV-1 TRANSCRIPTIONAL LANDSCAPE IN ART-TREATED PEOPLE WITH HIV

**FIGURE 1:**
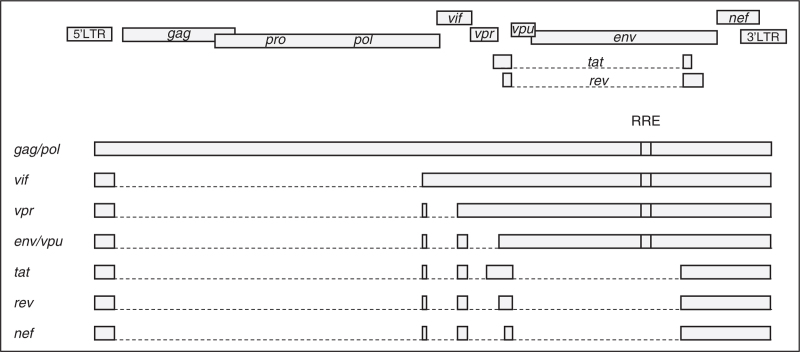
A simplified schematic representation of HIV-1 splicing. Upper panels: HIV-1 open reading frames. Lower panels: the main representatives of unspliced, incompletely spliced, and multiply spliced HIV-1 RNA classes. Gag and Pol proteins are expressed from the 9 kb unspliced RNA. The other HIV-1 proteins are expressed from either incompletely (Vif, Vpr, Env, and Vpu) or multiply (Tat, Rev, and Nef) spliced RNAs. Rev response element (RRE) is present in unspliced and incompletely spliced RNAs but not in multiply spliced RNAs. Exons are shown by bars and introns by dashed lines. (Figure adapted from [37]; reproduced with permission from Elsevier.).

HIV-1 infection produces more than 100 different viral transcripts, all derived by alternative splicing from the full-length unspliced (US) RNA that is transcribed from the integrated provirus (Fig. 1) [34,35]. HIV-1 splicing has been covered in depth by recent reviews [36,37]. Upon proviral integration, only short (2 kb) multiply spliced (MS) transcripts are initially produced that encode the regulatory proteins Tat, Rev, and Nef. As the infection progresses, a shift can be observed towards the production of 9 kb US and 4 kb incompletely spliced transcripts that encode the structural and accessory proteins Gag, Pol, Env, Vif, Vpr, and Vpu [38,39]. This shift is dependent on a threshold level of the Rev protein, which facilitates the export of the US and incompletely spliced RNA from the nucleus by binding to the Rev response element (RRE), a stem-loop structure located in the *env* open reading frame of these RNAs [40,41]. Due to the relative inefficiency of HIV-1 splicing and to the mechanism of nuclear export of US RNA, US RNA significantly outnumbers incompletely spliced and MS RNA in PWH, in both untreated and treated infection [26,27,42,43,44^▪▪^,^45^,^46^]. Moreover, MS RNA decays much faster and to a larger extent than US RNA upon ART initiation [44^▪▪^,^47^–^49^]. On top of this, while almost every HIV-1 provirus can produce some short abortive transcripts, the copy numbers of US transcripts that are elongated, completed, and polyadenylated are much lower. In fact, the Yukl group discovered a reverse correlation between the size of an HIV US RNA transcript and its abundance [42,50]. This can be due to specific latency blocks to transcriptional elongation, completion, and splicing [42], premature termination of RNA polymerase II transcription during elongation [51,52], reduced stability of extended transcripts, preferential immune clearance of cells containing extended HIV-1 transcripts, or to a combination of these effects.

More than 90% of infected cells in ART-treated PWH harbor genetically defective proviruses [53–56,57^▪^]. These proviruses exhibit various defects, such as large internal deletions, hypermutation, or mutation of essential motifs like the packaging signal/major splice donor, rendering them unable to cause viral rebound. Despite this, some of these defective proviruses can produce viral (or novel) transcripts and sometimes viral proteins as well [58^▪^,^59^,^60^]. This is possible because ART only prevents infection of new cells but does not inhibit viral RNA transcription or translation in cells that were infected prior to ART initiation, or in the progeny of these cells.

As a result of the scarcity of genetically intact HIV-1 proviruses, combined with the low abundance of full-length US RNA, intact full-length US transcripts are rare in ART-treated PWH. Martin *et al.*[57^▪^] recently developed a novel intact viral RNA assay (IVRA), based on the principle of the intact proviral DNA assay (IPDA) [61], to quantify likely intact, 3’ defective, and 5’ defective HIV-1 RNA. Using this assay, they detected a strong excess of the 3’ defective over the 5’ defective transcripts, confirming the previous finding that longer US transcripts are much less abundant than the shorter [42]. Moreover, only a median of <1% of the total CA US RNA was found to be intact in this assay [57^▪^]. These results were confirmed by two recent publications from the Imamichi group that used near-full-length (NFL) single-genome and single-transcript sequencing of CA DNA and US RNA from ART-treated PWH [58^▪^,62^▪^]. In these studies, 99.8% and 100% of amplified transcripts, respectively, were found to be defective, with the defective phenotype being nearly always due to large internal deletions. It must be noted that NFL sequencing methods are notoriously inefficient and may miss the majority of full-length proviruses (or transcripts arising from these proviruses) due to frequent amplification failures, as recently demonstrated [63]. In contrast, proviruses or transcripts with large internal deletions are amplified much more efficiently, resulting in a bias of NFL sequencing-based methods towards amplification of short, defective proviruses or transcripts. However, the proportions of intact proviruses among total proviruses in these NFL studies [58^▪^,62^▪^] were higher than the proportions of intact transcripts among total transcripts, again in line with the lower abundance of the longer transcripts [42].

Importantly, these studies only assessed the proportions of intact vs. defective transcripts, but did not address the relative transcriptional activity of intact vs. defective proviruses. Answering this question requires technically demanding assays, as it necessitates a combined assessment of proviral intactness and transcriptional activity in the same single cell. A recent study used multiplexed single-cell RNAflow-fluorescence in situ hybridization (FISH) to identify HIV-infected cells that express viral RNA during ART [33^▪▪^]. They found all HIV-1 proviruses sequenced from HIV RNA+ cells to be defective, although the number of sequenced NFL proviral amplicons in this study was limited. This confirms the earlier data from the same group, obtained using a similar methodology, that intact proviruses are rarely present in cells expressing viral RNA upon *ex vivo* stimulation [64].

Einkauf *et al.*[32^▪▪^] developed PRIP-seq, a limiting dilution-based assay that simultaneously captures the proviral sequence, the corresponding chromosomal integration site, and the level of HIV-1 RNA expression of in single infected cells. Overall, they scored similar proportions (26.7% and 33.6%) of intact and defective proviruses in ART-treated PWH that were transcriptionally active. However, these numbers only refer to the fractions of proviruses that could transcribe “long LTR” HIV-1 RNA. This “long LTR” assay detects all 5’ long terminal repeat (LTR)-containing transcripts that are at least ∼180 nt long, and considering the frequent termination of US RNA transcription in ART-treated PWH [42], most of these transcripts are expected to be short. Although the Einkauf study did not specifically report the frequencies of cells containing completed US RNA, it is plausible that full-length intact US transcripts would be rare because in most cases, transcription from an intact provirus is expected to terminate prior to completion.

In summary, there is evidence from several studies that, although US RNA is abundantly expressed in ART-treated PWH and the rates of transcription initiation and proximal elongation seem to be similar between intact and defective proviruses, intact full-length US transcripts are rare and most US RNA is derived from defective proviruses. Indeed, initiated, elongated, and completed US transcripts have been shown to correlate with total (which is mostly defective) but not with intact HIV-1 DNA [65]. As the *tat*/*rev* coding region is frequently deleted in defective proviruses [61], these proviruses are not expected to transcribe high levels of US RNA in the absence of the HIV-1 Tat protein. Also, depending on the presence of splice sites in the defective US transcript, its nuclear export might be impeded due to the absence of the viral Rev protein.

On the contrary, MS RNA production requires the presence of several intact genomic regions, such as splice sites or exonic splicing enhancers, and it is challenging to detect MS RNA in PWH on long-term suppressive ART without *ex vivo* cellular stimulation. Therefore, it is plausible that US RNA is derived from defective proviruses more frequently than MS RNA, and MS RNA transcription competence may be a more proximal surrogate of proviral replication competence compared to US RNA transcription competence [20,66]. Indeed, MS RNA measured after *ex vivo* stimulation of ART-treated PWH-derived CD4+ cells correlated much stronger than US RNA with either the virus release into culture supernatant or the frequency of CD4+ cells that harbor replication-competent HIV-1 measured by quantitative viral outgrowth assay (QVOA) [67,68]. However, MS RNA did not correlate with intact HIV-1 DNA in the study by Tumpach *et al.*[65].

## THE ROLE OF PERSISTENT HIV-1 TRANSCRIPTION IN RESIDUAL VIRAL PATHOGENESIS UNDER ART

It has been firmly established that ART-treated PWH experience persistent inflammation and chronic immune activation despite effective therapy, leading to the development of non-AIDS co-morbidities, mainly cardiovascular disease or metabolic syndrome [69]. The defective proviruses that are transcription- and translation-competent, previously considered irrelevant, are increasingly being linked to residual HIV-1 pathogenesis under suppressive ART [70,71]. Several (but not all) studies indicated an association between the CA RNA levels and residual immune activation and inflammation in ART-treated PWH [72–74]. We longitudinally measured more than 50 virological and immunological biomarkers during the first 96 weeks of ART and demonstrated that the CA HIV-1 US/MS RNA ratio, measured at 12 weeks of ART, was negatively predictive of the immunological response to ART (normalization of the CD4^+^ T-cell count) at 48 and 96 weeks [44^▪▪^]. Moreover, the US/MS RNA ratio was positively associated with markers of CD4^+^ T-cell activation and apoptosis at 12 weeks of ART, but still outperformed these host markers in the prediction of immunological response to therapy [44^▪▪^]. The fact that a virological biomarker performed better than any immunological biomarker in predicting an immunological outcome highlights the importance of considering the residual HIV-1 activity on ART as a correlate and a possible cause of the residual immune dysfunction that frequently occurs despite virologically suppressive ART. Very recently, the Imamichi group also evaluated the predictive markers of the immunological response to ART [62^▪^]. Confirming our observations, they described an association between the US RNA copy number (all sequenced US RNA was derived from defective proviruses in their study) and the immunological nonresponse. As immunological nonresponders present a higher risk of developing AIDS- and non-AIDS-related morbidity and mortality [75–77], these results link the expression of the defective proviruses to the residual HIV-1 pathogenesis under ART.

In another recent publication, the same group [58^▪^] demonstrated a strong association between CA US RNA (which was overwhelmingly defective) and the western blot score (measure of anti-HIV-1 antibodies as a surrogate marker for viral protein expression). They also revealed the long persistence of multiple transcriptionally active defective proviral clones and reported a positive correlation between either total HIV-1 DNA or US RNA and the CD8^+^ T-cell count [58^▪^], which is in line with the results of an earlier study that demonstrated that production of HIV-1 antigens from defective proviruses can be recognized by CD8^+^ cytotoxic T lymphocytes [78]. In the same vein, two recent publications in Cell Host & Microbe addressed the impact of persistent HIV-1 transcription during ART on HIV-1-specific CD4+ and CD8^+^ T-cell responses [33^▪▪^,79^▪^]. Takata *et al.* investigated a cohort of PWH after 2 years of ART initiated during acute or chronic HIV-1 infection and found that the magnitude of CD8^+^ T cells specific for HIV-1 Gag, Pol, Nef, and Vif proteins positively associated with CA RNA and that high total HIV-1 DNA levels strongly associated with the maintenance of short-lived HIV-specific CD8^+^ T cells. This suggests that persistent HIV-1 transcription under ART maintains the magnitude of the HIV-specific CD8^+^ T-cell response but prevents their differentiation into functional cells. The authors concluded that targeting the persistent transcription could improve HIV-1-specific CD8+ T-cell functionality needed for HIV-1 remission [79^▪^]. Dubé *et al.* found that, despite being derived from defective proviruses, CA RNA under ART positively correlated with total HIV-specific CD4^+^ and CD8^+^ T-cell responses and with multiple HIV-specific T-cell clusters, suggesting that the persistent HIV-1 transcription maintains the HIV-specific T-cell responses during suppressive ART. These associations were particularly strong for the CA-p24-expressing cells [33^▪▪^].

Taken together, these results add to the growing body of evidence that persistent defective proviruses, especially those that are transcriptionally active, are not genetic ‘junk’ that is irrelevant for HIV-1 pathogenesis and cure. Rather, these results suggest a continuous crosstalk between the residual HIV-1 gene expression under ART and the immune system. Recent studies suggest that transcriptionally active intact proviruses are actively selected against, presumably by immune-mediated surveillance mechanisms, during prolonged ART and natural HIV-1 control [32^▪▪^,80^▪^,^81^]. In the recent years, it has become clear that immune clearance plays a surprisingly prominent role in shaping the reservoir [82]. It is plausible that in most PWH, the process of ‘natural HIV cure’ starts from the first day of ART, as the immune system is continuously removing the replication-competent proviruses and selecting for intact proviruses that are in the state of ‘deep latency’ [23]. Interestingly, not only intact [56,83–85], but also transcription-competent defective proviruses have been shown to decay with time on ART [32^▪▪^], suggesting that these proviruses are subject to the same immune selection forces. Despite this, in many PWH, expanded clones of transcriptionally active proviruses persist for years and may even cause a persistent nonsuppressible plasma viremia [86,87]. Future research should decipher the precise mechanisms that allow intact and defective proviruses expressing HIV-1 RNA and proteins to avoid elimination by the host immunity, but in the recent years it has become clear that HIV-1 can persist not only by transcriptional latency, but also by resistance to the immune clearance of HIV-expressing cells [21,88,89,90^▪^]. Possible mechanisms of this immune resistance include the capability of the viral protein Nef, encoded by many defective proviruses [56,58^▪^], to downregulate MHC-I [91^▪▪^].

## SHOULD WE REASSESS THE SIGNIFICANCE OF PERSISTENT HIV-1 TRANSCRIPTION UNDER ART?

Despite the effectiveness and improved accessibility of modern ART, a quarter of PWH still do not have access to the therapy. The lifelong treatment is also not without its negative effects and complexities, such as drug toxicities, medication interactions and polypharmacy, adherence issues, stigma, and the overall financial burden on PWH and the healthcare system. Therefore, finding a cure for HIV-1 remains a global priority. There are two main reasons why persistent HIV-1 transcription under ART, despite being mainly derived from defective proviruses, is relevant for the long-term health of PWH and the cure research. First, if transcriptionally active defective proviruses are indeed pathogenic, this should change the concept of HIV-1 cure. The current understanding of HIV-1 cure is eradication or suppression of replication-competent proviruses only, in order to prevent viral rebound upon stopping ART. However, if transcriptionally active defective proviruses can contribute to chronic immune activation, inflammation, and immunosenescence that are frequently observed in PWH on suppressive ART [92,93], then a broader definition of an HIV-1 cure should include eradication, suppression, or inactivation of all transcription-competent, including genetically defective, proviruses.

A second reason why persistent HIV-1 transcription is relevant for the cure research is because it predicts viral rebound upon stopping ART. Posttreatment controllers (PTCs), rare individuals who can control HIV-1 replication after ART interruption, are considered a model for HIV-1 remission [94–96], and the time to viral rebound after treatment interruption (TI) has been proposed to correlate with the replication-competent reservoir size [97]. Therefore, identifying robust predictors of time to rebound is an important priority for the cure research. Remarkably, a number of groups have shown that CA US HIV-1 RNA, measured prior to TI, is a powerful predictor of time to viral rebound [98–102]. We demonstrated that US RNA was predictive not only of the time to viral rebound to both >50 and >400 copies/ml but also of the magnitude of the viral rebound, independently of pre-ART virological biomarkers [100]. Recently, Wedrychowski *et al.*[103^▪^] reported lower levels of initiated, elongated, and completed US RNA (but not MS RNA), measured pre-TI, in future PTCs, as compared to noncontrollers. Taken together, these results create an apparent paradox: why does US RNA predict viral rebound if the majority of US transcripts is derived from defective proviruses?

One possible answer to this question is that the level of total (mostly defective) HIV-1 transcription may correlate with the level of transcription from intact proviruses. In individuals on prolonged ART and elite controllers, intact HIV-1 proviruses are enriched for nongenic chromosomal positions and other features of ‘deep latency’ that suppress proviral transcription [32^▪▪^,80^▪^,^81^,^104^]. Therefore, for the replication competence, the transcription competence of a provirus, influenced by its relative position in the nuclear 3D chromatin architecture [105,106], is likely as important as its genetic intactness.

Another possible explanation for the predictive value of on-ART US RNA for the viral rebound upon TI is that persistent HIV-1 transcription level in ART-treated PWH reflects the host immune pressure that shapes the viral rebound if ART is stopped. In the recent years, it has become apparent that rebound viruses detected in plasma post-TI are mostly phylogenetically distinct from preinterruption replication-competent or genetically intact proviruses identified by QVOA or NFL sequencing, respectively [107–109]. This suggests that the reactivation potentials of individual proviruses may differ *in vivo* and *ex vivo*, due to the immune-mediated selection of rebound viruses *in vivo*. Two recent studies indicated that not all replication-competent reservoirs (as assayed by QVOA) are rebound-competent, as both innate and adaptive immunity were implicated in the control of the rebound. Bertagnolli *et al.*[110] demonstrated that the outgrowth of a substantial fraction of reservoir viruses was blocked by autologous IgG antibodies against HIV-1 envelope, and Gondim *et al.*[111] reported that HIV-1 isolates detected during TI were resistant to type-I interferons. This suggests that the viral rebound is determined not only by the number of the intact proviruses and their chromatin context, but also by the strength of the anti-HIV host immune response (innate and/or adaptive) that restricts the rebounding virus.

The level of US RNA, despite it being mostly derived from defective proviruses, may also be influenced by this immune response already under ART. There is ample evidence that suggests that production of HIV-1 (or novel) transcripts and antigens from defective proviruses can be recognized by both innate and adaptive immunity [33^▪▪^,^44^^▪▪^,58^▪^,^78^,79^▪^,^112^,^113^]. Experimental depletion of CD8^+^ T cells in simian immunodeficiency virus (SIV)-infected and ART-treated rhesus macaques resulted in an increase in plasma viremia in all animals and repopulation of CD8^+^ T cells was associated with prompt reestablishment of virus control [114]. These results suggest a role of CD8^+^ T cells in controlling viral production during ART. As CD8^+^ cell depletion did not induce any significant change in the number of SIV DNA-positive CD4^+^ T cells in that study, it is plausible that the effect of the depletion on plasma viremia was mediated by an increased SIV transcription level per provirus. The effect of CD8^+^ T-cell depletion on the virus production was recently confirmed by another study in SIV-infected rhesus macaques and HIV-infected humanized mice [115]. Apart from increased plasma viremia, this study detected a statistically significant increase in the percentage of SIV RNA^+^ cells with high levels of SIV RNA in the lymph node after CD8^+^ cell depletion.

Taken together, these results suggest that the persistent HIV-1 transcription level under ART is an indicator of the strength of the host antiviral immune response that influences the viral rebound upon TI. In particular, HIV-1 transcription level may indicate the level of T-cell exhaustion. We recently observed that HIV-1 US RNA and especially the level of US RNA transcription per provirus (US RNA/total DNA ratio) strongly positively correlated with the percentages of CD4^+^ cells expressing exhaustion markers CTLA-4 and PD-1 during ART [44^▪▪^]. As a state of T-cell exhaustion is marked by an ineffective antiviral immune response and exhausted T cells are unable to exert antiviral effector functions [116], this suggests one possible explanation for the predictive value of pre-TI US RNA for the time to rebound. This and other recent findings discussed above suggest that HIV-1 transcription should be assessed in the cure-directed clinical trials [117]. For example, it could be used as a marker to evaluate the efficacy of immune interventions aimed at reducing the reservoirs. In general, whereas MS RNA is a better correlate of the replication-competent reservoir size than US RNA, US RNA may be a better indicator of the *in vivo* rebound competence that is shaped by the antiviral immune responses. Thus, both US and MS RNA have to be considered for a complete assessment of the functional HIV-1 reservoir.

## CONCLUSION

There is increasing evidence that persistent HIV-1 transcription may be a major contributor to chronic inflammation and immune activation during ART and can therefore play an important role in the pathophysiology of treated PWH. Further studies should provide a definitive proof of the pathogenicity of transcriptionally active defective proviruses and inform the design of novel therapeutics to suppress their activity. In particular, the ‘block and lock’ approach [118,119] can be used as permanent adjuvant therapy to conventional ART in order to counteract residual HIV-1 pathogenesis. Obefazimod (ABX464) was recently shown to reduce both total HIV-1 DNA and the level of transcription initiation per provirus [120]. However, another recent study failed to confirm the effect of this drug on HIV-1 transcription, although the effect on total DNA was still observed [121]. This and other recently identified drugs that target viral transcription or RNA processing [122,123] have the potential to suppress immune activation and dysfunction, facilitate immune reconstitution on ART, and possibly contribute to the HIV-1 cure. The significance of persistent HIV-1 transcription during ART for the cure research should be reassessed in light of the recent data suggesting its role as an indicator of the antiviral immune response that is shaping the reservoir and rebound.

## Acknowledgements

*None.*

### Financial support and sponsorship

*This work was supported by the grant no. 09120011910035 from the Dutch Medical Research Council (ZonMw). Céline Fombellida-Lopez is a FRIA grantee (grant no. 40014489) of the Belgian Fund for Scientific Research (Fonds de la Recherche Scientifique – FNRS).*

### Conflicts of interest

*There are no conflicts of interest.*

## REFERENCES AND RECOMMENDED READING

Papers of particular interest, published within the annual period of review, have been highlighted as:

▪ of special interest
▪▪ of outstanding interest
